# Performance of the IMPACT and Helsinki models for predicting 6-month outcomes in a cohort of patients with traumatic brain injury undergoing cranial surgery

**DOI:** 10.3389/fneur.2022.1031865

**Published:** 2022-10-31

**Authors:** Lei Chen, Haiting Xu, Jianqing He, Chunlei Zhang, Andrew I. R. Maas, Daan Nieboer, Rahul Raj, Hong Sun, Yuhai Wang

**Affiliations:** ^1^Department of Neurosurgery, Wuxi Clinical Hospital, Anhui Medical University (The 904th Hospital of PLA), Wuxi, China; ^2^Department of Emergency, Joint Logistics Support Unit No. 904 Hospital, Wuxi, China; ^3^Department of Neurosurgery, Neurotrauma Centre of PLA, Joint Logistics Support Unit No. 904 Hospital, Wuxi, China; ^4^Department of Neurosurgery, Antwerp University Hospital and University of Antwerp, Edegem, Belgium; ^5^Department of Public Health, Erasmus MC, Rotterdam, Netherlands; ^6^Department of Neurosurgery, Helsinki University Hospital, Helsinki, Finland

**Keywords:** external validation, outcome, prognostic model, Helsinki CT, traumatic brain injury

## Abstract

**Background and aim:**

Prediction models for patients with traumatic brain injury (TBI) require generalizability and should apply to different settings. We aimed to validate the IMPACT and Helsinki prognostic models in patients with TBI who underwent cranial surgery in a Chinese center.

**Methods:**

This validation study included 607 surgical patients with moderate to severe TBI (Glasgow Coma Scale [GCS] score ≤12) who were consecutively admitted to the Neurotrauma Center of People's Liberation Army (PLANC), China, between 2009 and 2021. The IMPACT models (core, extended and lab) and the Helsinki CT clinical model were used to estimate 6-month mortality and unfavorable outcomes. To assess performance, we studied discrimination and calibration.

**Results:**

In the PLANC database, the observed 6-month mortality rate was 28%, and the 6-month unfavorable outcome was 52%. Significant differences in case mix existed between the PLANC cohort and the development populations for the IMPACT and, to a lesser extent, for the Helsinki models. Discrimination of the IMPACT and Helsinki models was excellent, with most AUC values ≥0.80. The highest values were found for the IMPACT lab model (AUC 0.87) and the Helsinki CT clinical model (AUC 0.86) for the prediction of unfavorable outcomes. Overestimation was found for all models, but the degree of miscalibration was lower in the Helsinki CT clinical model.

**Conclusion:**

In our population of surgical TBI patients, the IMPACT and Helsinki CT clinical models demonstrated good performance, with excellent discrimination but suboptimal calibration. The good discrimination confirms the validity of the predictors, but the poorer calibration suggests a need to recalibrate the models to specific settings.

## Introduction

Traumatic brain injury (TBI) is a leading cause of mortality and disability in China, affecting up to 2% of the population per year (1, 2). A recent population-based study, extrapolating from data from China's disease surveillance points system, reported an age-adjusted mortality rate of ~13 per 100,000 population over 2013. The patient volume is very high in most Chinese centers, and a great need is felt for prognostic models for predicting outcome that apply to the setting in China. If applicable, these models could serve as a tool to evaluate healthcare levels of different healthcare institutions as a new plan for brain injury. Uncertainty, however, exists if models developed in other settings apply to the situation in China, where decompressive craniectomy is frequently performed in patients with more severe TBI (3). A relatively large number of prognostic models have been developed for TBI, but only two have been developed on large patient numbers and validated externally: the CRASH (Corticosteroid Randomization After Significant Head injury) (4) and IMPACT (5) (International Mission for Prognosis and Analysis of Clinical Trials in TBI) prognostic models. More recently, the Helsinki CT (computerized tomography) clinical model was proposed based on the Helsinki CT score and clinical parameters (6). Good performance was reported, but external validation in a new setting has not yet been performed. External validation is important to assess generalizability and to support the use of the models for a wide range of applications, since differences in treatment and health care organization between populations exist and may affect the performance of models (6, 7). In our setting, which serves as a regional referral center for neurotrauma, a large number of patients admitted for TBI are treated surgically either for evacuation of a mass lesion or for treatment of raised intracranial pressure (ICP) by decompressive craniectomy. For this validation study, we focused on the IMPACT prognostic models as they have been previously extensively validated and on the Helsinki CT clinical model, as the development population for this model is more recent and shows better comparability to our patient cohort than that of the IMPACT models.

This study aimed to explore the applicability of the IMPACT and Helsinki CT clinical models for the prediction of 6-month mortality and unfavorable outcomes in a cohort of TBI patients in China who underwent surgical treatment.

## Methods

### Study population

The study was conducted at the Neurotrauma Center of the People's Liberation Army (PLANC) in Wuxi, China, which provides emergency neurosurgical care for a regional population of approximately 6,500,000 people. The standard medical management of raised intracranial pressure (ICP) was based on the 2007 Brain Trauma Foundation guidelines (8). Data on patients with TBI are prospectively included in the ongoing PLANC database. Data collected included patient demographics (age, sex, and ethnicity), mechanism of injury (falls, road traffic incidents, assault and others), severity of injury (Glasgow Coma Scale [GCS] score, pupillary response, and presence of extracranial injuries), second insults (hypoxia, hypotension, and hypo/hyperthermia), lab tests (hemoglobin and glucose levels), brain CT characteristics, surgical therapy and 6-month outcome. Data on admission characteristics were collected before any hospital intervention. Outcome was assessed according to the Glasgow Outcome Scale (GOS) by a staff neurosurgeon during the follow-up visit (Appendix 1). The cohort consisted of 607 consecutive patients who had undergone cranial surgery for TBI between 2009 and 2021. The following inclusion criteria were applied: patients older than 14 years, with an admission GCS score ≤12, admitted within 8 h after injury. Additional radiological information, necessary to calculate the IMPACT models and the Helsinki CT score, was retrospectively obtained on central review by one of the authors (C.L.Z.) blinded to the outcome.

The local IRB of the People's Liberation Army (PLANC) Hospital in Wuxi approved the study and waived the need for obtaining informed consent, as the study is purely observational.

### Indications for surgical management

Surgical management of intracranial mass lesions conformed to published guidelines (9, 10). Within the hospital, a relatively liberal indication exists for performing a decompressive craniectomy. This was performed in the following situations: (1) After evacuation of a mass lesion (a large bony decompression usually performed within the confines of patient positioning), if the brain is swollen or ICP remains consistently above 20 mmHg after the bone flap is replaced. (2) as treatment of raised ICP, refractory to medical therapy.

Unilateral decompression was performed when the cerebral swelling was predominantly in one hemisphere; bifrontal decompression was performed when the cerebral swelling was distributed over both hemispheres.

### Prognostic models

The IMPACT prognostic models have been developed on large patient samples from multiple countries with state-of-the-art methodology and have been extensively validated externally (7, 11–15). The IMPACT models were developed on prospectively collected data from adult patients (age ≥14 years) with a GCS score of ≤12 who had been included in eight randomized controlled trials (RCTs) and three observational series (total *n* = 8,509) between 1984 and 1997 (5, 16). The IMPACT core model is based on three basic predictors: age, the GCS motor scale component and pupillary reactivity. The IMPACT extended model further includes the presence of second insults (hypoxia and hypotension), the Marshall CT classification, presence of an epidural hematoma (EDH) and presence of traumatic subarachnoid hemorrhage (tSAH) as additional predictors (5). The IMPACT lab model further adds admission glucose and hemoglobin concentrations. The more complex IMPACT models have been shown to have better discrimination.

The Helsinki CT clinical model was developed on patient data from an open-cohort retrospective study including 869 adults with traumatic brain injury aged 14 years or over (6). The Helsinki CT clinical model consists of the Helsinki CT score and three clinical variables (age, GCS motor scale component and pupillary reactivity) (http://links.lww.com/NEU/A676). The Helsinki CT score is based on four key variables: bleeding type and size, intraventricular hemorrhage, and status of suprasellar cisterns. In the development population, the Helsinki CT score showed good discrimination with an area under the receiver operating characteristic curve (AUC) of 0.74–0.75 for mortality and unfavorable outcomes. Combining the Helsinki CT score with clinical variables (i.e., Helsinki CT clinical model) increased the discriminative ability for mortality (non-significant) and significantly for unfavorable outcomes (6).

### Statistical analyses and outcome assessment

Missing values of baseline characteristics were statistically imputed by applying a multiple imputation procedure following the Hmisc function in R software (AregImpute function in the R Hmisc package). The highest percentage of missing values was for the variable glucose (5%; Supplementary Table S1). Imputation of baseline variables is considered preferable to complete case analysis (17). Patient characteristics were summarized descriptively, reporting the median (and interquartile range) for continuous variables and frequency (percentage) for categorical variables. Differences in characteristics between data sets were analyzed by the chi-square test and Student's *t*-test. The prognostic effects of parameters in the PLANC study were assessed in a multivariable logistic regression model, and their strength was expressed by the odds ratio (OR). The odds ratios obtained were compared to those previously reported in the analysis of IMPACT data (5).

We evaluated the performance of the models in terms of discrimination and calibration. Discrimination informs how well the models distinguish between the outcomes of interest. The discriminative ability of the models was evaluated by the AUC. An AUC equal to 0.5 indicates that the discriminative ability of the model is no better than a coin toss, and an AUC of 1 indicates perfect discrimination. An AUC≥ 0.8 is considered to represent adequate discriminative ability of the model. Differences in discriminatory performance between the IMPACT and Helsinki models were analyzed with the DeLong test (18). Calibration refers to the agreement between predicted and observed outcomes. Calibration was quantified using calibration-in-the-large and the calibration slope and was assessed visually using calibration plots. Calibration-in-the-large measures whether predicted risks are systematically too high or too low and ideally should be equal to zero (19). The calibration slope measures whether predictor effects are on average too strong or too weak and should ideally be equal to one. Furthermore, we calculated Nagelkerke *R*^2^ values as a measure of overall model performance. All statistical analyses were performed in R version 2.10 (R Foundation, Vienna, Austria).

## Results

Data were collected on 607 surgical patients with moderate to severe TBI admitted to our neurotrauma intensive care unit between 2009 and 2021 in Wuxi, China. Decompressive craniectomy was performed in 531 cases (87%). Of these, 449 patients (85%) underwent unilateral decompressive craniectomy, and 82 patients (15%) underwent bilateral decompressive craniectomy. Details on the surgical and CT characteristics of the PLANC cohort are described in Table 1.

**Table 1:** Surgical and computerized tomography characteristics of the PLANC cohorts^a^.

**Variable**	**PLANC (*n* = 607)**
**Surgical lesion;** ***n*** **(%)**	
Subdural hematoma	511 (85%)
Intracerebral hemorrhage	412(68%)
Epidural hematoma	145 (24%)
**Mass lesion size** **≥25 cm**^**3**^**;** ***n*** **(%)**	441 (72%)
**IVH; n(%)**	126 (21%)
**Suprasellar cisterns;** ***n*** **(%)**	
Normal	201 (33%)
Compressed	207 (34%)
Absent	199 (33%)

IVH, Intraventricular Hemorrhage.

### Comparison of cohorts

Table 2 presents the baseline characteristics of the PLANC cohort and compares these to the patient characteristics of the cohorts used to develop the IMPACT and the Helsinki CT clinical models. Compared to the IMPACT cohort, patients in the PLANC cohort were significantly older (median of 48 vs. 30 years), had a higher proportion of one non-reactive pupil (26 vs. 12%), higher proportions of hypoxia, hypotension, tSAH, EDH and more patients with a GCS motor score of 5 or 6 (50 vs. 30%). No patients in PLANC had a CT classification of I (no visible damage) or II (abnormalities present, but no mass lesion or signs of raised intracranial pressure) vs. 42% in IMPACT. The percentage of patients in CT Class V/VI (mass lesion) was nearly twice as high in PLANC compared to IMPACT (70 vs. 38%). The frequency of the 6-month vegetative state was higher in the PLANC cohort than in the IMPACT cohort (12 vs. 4%, *p* < 0.05), as was the proportion of unfavorable outcomes (52 vs. 48%).

**Table 2:** Characteristics of patients in IMPACT study (no imputation of missing values), Helsinki and PLANC cohorts.

**Characteristics**	**Coding**	**PLANC**	**IMPACT**		**Helsinki**	
		**(*n* = 607)**	**(*n* = 8509)**	*p*-value*	**(*n* = 869)**	*p*-value**
**Age (years)**	Median	48(38–60)	30 (21–45)*	*p* < 0.001	57 (43-68)	*p* < 0.001
	(25–75 percentile)					
**Motor score**, ***n*** **(%)**	None (1)	84 (14%)	1395 (16%)	*p* < 0.001	141 (16%)	*p* < 0.001
	Extension (2)	104 (17%)	1042 (12%)		42 (5%)	
	Abnormal flexion (3)	83 (14%)	1085 (13%)		36 (4%)	
	Normal flexion (4)	28 (5%)	1940 (23%)		94 (10%)	
	Localizes(5) /obeys (6)	306 (50%)	2591 (30%)		556 (64%)	
**Pupillary**	Both pupils reactive	298 (49%)	4486 (63%)	*p* < 0.001	644(74%)	*p* < 0.001
**Reactivity**, ***n*** **(%)**	One pupil reactive	156 (26%)	886 (12%)		101 (12%)	
	No pupil reactive	148 (24%)	1754 (25%)		124 (14%)	
**Hypoxia**, ***n*** **(%)**	No	475 (78%)	4336 (51%)	*p* = 0.894	737 (85%)	*p* < 0.01
	Yes or suspected	124 (20%)	1116(20%)		132 (15%)	
**Hypotension**, ***n*** **(%)**	No	548(90%)	5249 (62)%*	*p* < 0.001	803(92%)	*p* = 0.21
	Yes or suspected	57 (9%)	1171 (18%)		66 (8%)	
**Marshall CT**	I	0	360 (7%)	*p* < 0.001	-	*p* < 0.001
**Classification**, ***n*** **(%)**^a^	II	0	1838 (35%)		282 (33%)	
	III	62 (10%)	863(17%)*		37 (4%)	
	IV	115 (19%)	187 (4%)		30 (4%)	
	EML/NEML	427 (70%)	1944 (38%)		520 (60%)	
**tSAH**, ***n*** **(%)**^b^	No	26 (4%)	4080 (48%)*	*p* < 0.001	365(42%)	*p* < 0.001
	Yes or suspected	578 (95%)	3313 (45%)		504 (58%)	
**EDH**, ***n*** **(%)**^c^	No	459(76%)	6410 (75%)*	*p* < 0.001	779(90%)	*p* < 0.001
	Yes or suspected	145(24%)	999 (13%)		90 (10%)	
**Glucose (mmol/L)**	Median	8.5(6.5–11.4)	8.2 (6.7–10.4)*	*p* < 0.001	7.3 (6.1–8.9)	*p* < 0.001
	(25–75 percentile)					
**Hb (g/dL)**	Median	12.4 (11.7–13.4)	12.7(10.8–14.3)	*p* < 0.001	12.5(11.1-14.9)	*p* = 0.530
	(25–75 percentile)					
**6-month outcome** ^d^	Dead	170 (28%)	2396 (28%)		219(25%)	
	Vegetative	75 (12%)	351 (4%)		195(23%)	
	Severe disability	70 (12%)	1335 (16%)			
	Moderate disability	113 (19%)	1666 (20%)		455(52%)	
	Good recovery	179 (29%)	2761 (32%)			

*p*-values were calculated by either chi-square tests or student t-test.

* Comparison between PLANC and IMPACT

** Comparison between PLANC and Helsinki CT.

a Marshall CT classification: I, no visible intracranial pathology; II, midline shift 0–5 mm; III, cistems compressed or absent with midline shift 0–5 mm; IV, midline shift>5mm, EML, any lesion surgically evacuated; NEML, high-or mixed-density lession>25 mm, not surgically evacuated. EML and NEML were combined in IMPACT and Helsinki cohorts.

b tSAH, Traumatic subarachnoid hemorrhage.

c EDH, Epidural hemorrhage.

d 6-month outcome in survivors was classified into two categories in the Helsinki cohort (favorable and unfavorable outcome, dead and alive).

Compared to the Helsinki cohort, patients in PLANC were younger (median 48 vs. 57 years), less often had a GCS motor score of 5 and 6 (50 vs. 64%), but more often had one or two non-reactive pupils (26, 24 vs. 12, 14%, respectively; *p* < 0.001). The percentage of patients with hypoxia, tSAH, and EDH was higher in the PLANC cohort. A difference existed in the percentage of patients in CT Class V/VI (mass lesion) between PLANC and Helsinki (70 vs. 60%), but this was less pronounced than for IMPACT (38%).

### Predictor effects

A detailed overview of the associations between baseline characteristics and 6-month GOS is presented in Supplementary Table S2. Compared to IMPACT, PLANC patients with a GCS motor scale of 1–3, with absent pupillary activity, hypoxia, and hypotension had a poorer outcome. Compared to Helsinki, those with an absent motor response, hypoxia, hypotension or an EDH had poorer outcomes in PLANC. A detailed comparison of predictor effects between the IMPACT and PLANC cohorts is shown in Table 3. The effects of absent motor score, EDH, hypotension and glucose were all stronger in PLANC, whereas that of age was weaker in PLANC. The predictor effects of hemoglobin were in the opposite direction to those described for IMPACT.

**Table 3:** Predictor effects in IMPACT (*n* = 8,509) and PLANC cohorts (*n* = 607) (missing values were imputed).

		**IMPACT** * **Odds ratios (95% CI)**					**PLANC database Odds ratios (95% CI)**				
**Characteristics**	**Measure or category**	***N***	**Univariate**	**Core model**	**Extended model**	**Lab model**	***N***	**Univariate**	**Core model**	**Extended model**	**Lab model**
				***n* = 8509**	***n* = 6999**	***n* = 3554**			***n* = 607**	***n* = 607**	***n* = 607**
Age (years)	Median (25–75 percentile)	8,509	2.2 (2.0–2.3)	2.4 (2.2–2.5)	2.2 (2.0–2.3)	1.9 (1.7–2.1)	607	1.4(1.1–1.8)	1.5 (1.1–2.0)	1.4(1.0–1.9)	1.1(0.8–1.6)
Motor score of	None (1)	1,395	4.9 (4.3–5.5)	3.9 (3.4–4.5)	3.4 (2.9–4.0)	2.8 (2.1–3.7)	84	18.8(10.4–34.1)	11.9 (5.7–25.2)	8.9(4.1–19.7)	7.5 (3.0–19.1)
GCS	Extension (2)	1,042	7.2 (6.3–8.3)	5.7 (4.9–6.6)	4.6 (3.9–5.4)	4.3 (3.5–5.4)	104	11.6 (6.7–20.0)	7.3 (3.6–14.8)	6.2 (3.0–12.7)	5.3 (2.2–12.6)
	Abnormal flexion (3)	1,085	3.5 (3.1–4.0)	3.0 (2.6–3.5)	2.8 (2.4–3.2)	2.7 (2.2–3.3)	83	3.4 (1.8–6.3)	2.3 (1.1 −4.8)	1.9 (0.9–4.0)	1.5 (0.6–3.5)
	Normal flexion (4)	1,940	1.8 (1.6–2.0)	1.7 (1.5–1.9)	1.6 (1.4–1.8)	1.5 (1.3–1.8)	28	4.7 (1.9–11.4)	3.8 (1.5– 9.5)	2.8 (1.1–7.4)	2.8 (0.9–8.3)
	Localizes(5)/obeys(6)	2,591	1.0 (ref)	1.0 (ref)	1.0 (ref)	1.0 (ref)	306	1.0 (ref)	1.0 (ref)	1.0 (ref)	1.0 (ref)
Pupillary reactivity	Both pupils reactive	4,486	1.0 (ref)	1.0 (ref)	1.0 (ref)	1.0 (ref)	298	1.0 (ref)	1.0 (ref)	1.0 (ref)	1.0 (ref)
	One pupil reactive	886	2.7 (2.4–3.1)	1.8 (1.6–2.1)	1.6 (1.4–1.8)	1.4 (1.1–1.7)	156	3.4 (2.1–5.6)	1.6 (0.9–2.9)	1.6 (0.9–3.0)	1.2 (0.6–2.4)
	No pupil reactive	1,754	5.9 (5.3–6.6)	3.3 (3.0–3.7)	2.7 (2.4–3.1)	2.1 (1.6–2.6)	148	9.5 (5.9–15.3)	2.0 (1.0–4.0)	1.8 (0.9–3.7)	1.1(0.5–2.6)
Hypoxia	No	4,336	1.0 (ref)	–	1.0 (ref)	1.0 (ref)	475	1.0 (ref)	–	1.0 (ref)	1.0 (ref)
	Yes or suspected	1,116	2.1 (1.9–2.4)	–	1.3 (1.1–1.5)	1.4 (1.2–1.7)	124	2.9 (1.9–4.4)	–	1.0 (0.6–1.7)	0.8 (0.4–1.5)
Hypotension	No	5,249	1.0 (ref)	–	1.0 (ref)	1.0 (ref)	548	1.0 (ref)	–	1.0 (ref)	1.0 (ref)
	Yes or suspected	1,171	2.7 (2.4–3.1)	–	1.8 (1.6–2.1)	1.5 (1.2–1.8)	57	8.4 (4.5–15.4)	–	5.6 (2.7–11.4)	9.3 (3.7–23.6)
CT Classification	I	360	0.4 (0.3–0.5)	–	0.6 (0.5–0.8)	0.7 (0.5–0.9)	–	–	–	–	–
	II	1,838	1.0 (ref)	–	1.0 (ref)	1.0 (ref)	–	–	–	–	–
	III	863	2.6 (2.3–3)	–	1.7 (1.5–2.0)	1.7 (1.4–2.0)	62	1.0 (ref)	–	1.0 (ref)	1.0 (ref)
	IV	187	–	–	–	–	115	–	–	–	–
	EML/NEML	1,944	2.3 (2.0–2.6)	–	1.6 (1.4–1.9)	1.8 (1.5–2.2)	427	0.7 (0.5–1.1)	–	0.8 (0.5–1.2)	0.8 (0.5–1.4)
tSAH	No	4,080	1.0 (ref)	–	1.0 (ref)	1.0 (ref)	26	1.0 (ref)	–	1.0 (ref)	1.0 (ref)
	Yes or suspected	3,313	2.6 (2.4–2.9)	–	1.7 (1.5–1.8)	1.8 (1.6–2.1)	578	10.2 (1.4–76.2)	–	1.9(0.2–14.9)	2.0 (0.2–17.1)
Epidural hemorrhage	No	6,410	1.0 (ref)	–	1.0 (ref)	1.0 (ref)	459	1.0 (ref)	–	1.0 (ref)	1.0 (ref)
	Yes or suspected	999	0.6 (0.6–0.7)	–	0.6 (0.5–0.7)	0.6 (0.5–0.7)	145	0.3 (0.2–0.5)	–	0.4 (0.2–0.8)	0.4 (0.2–0.9)
Glucose (mmol/L)	Median (25–75 percentile)	4,830	1.7 (1.6–1.8)	–	–	1.3 (1.2–1.4)	576	.	–	–	5.6 (3.8–8.2)
Hb (g/dL)	Median (25–75 percentile)	4,376	0.7 (0.6–0.7)	–	–	0.8(0.70–0.9)	601	1.4 (1.1–1.8)	–	–	1.7 (1.3–2.3)

* predictor effects in IMPACT as reported by Steyerberg et al. (5).

### Prognostic model performance

#### Discrimination

**Figure 1:**
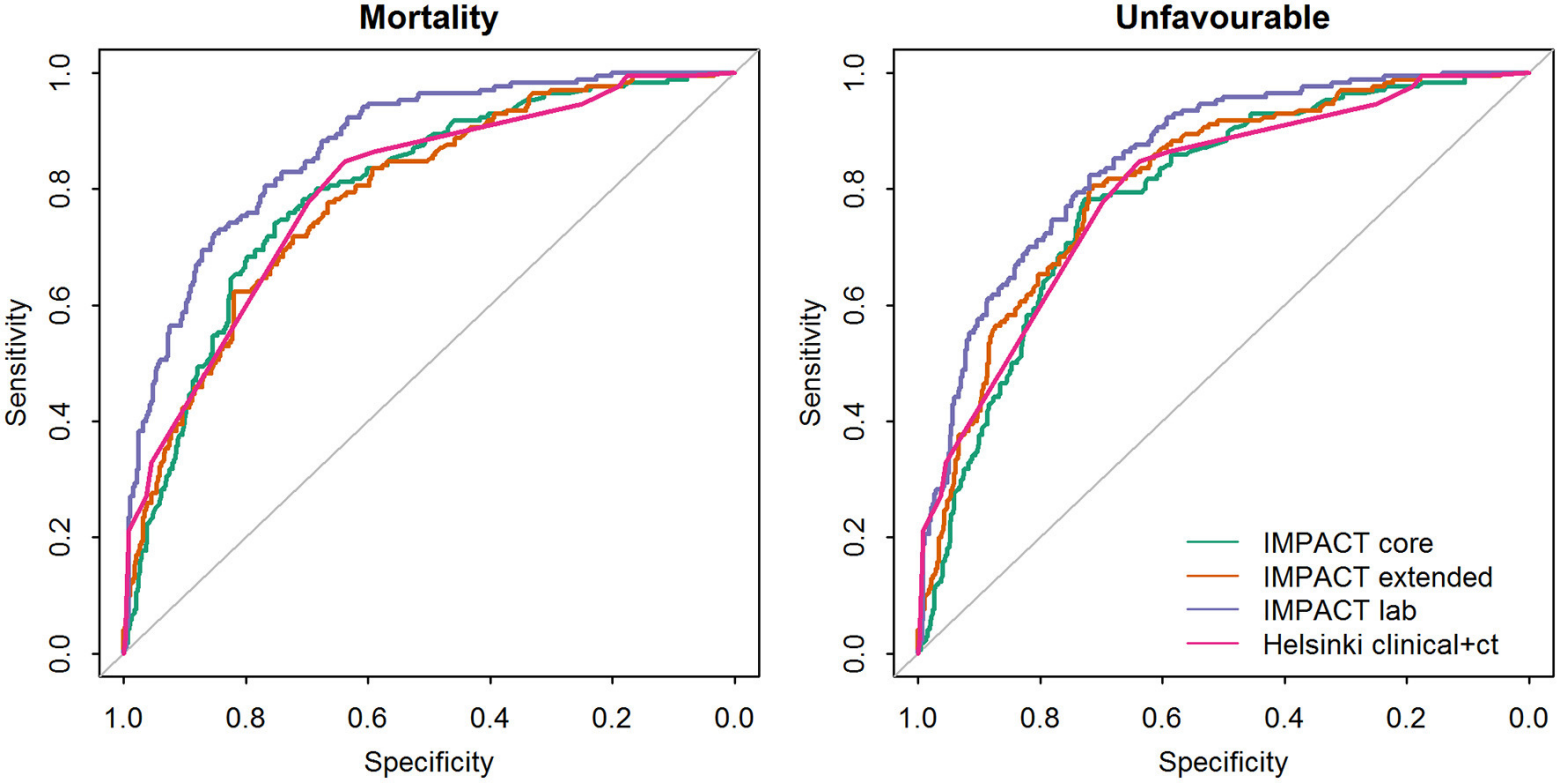
Comparison of the areas under the receiver operating characteristic curve (AUC's) between the prognostic models validated on the PLANC cohort.

The IMPACT models and Helsinki CT clinical model showed good ability to discriminate between survival and death and between favorable and unfavorable outcomes (Table 4, Figure 1), with AUC values mostly over 0.8. Higher values were achieved for the prediction of unfavorable outcomes than for mortality in nearly all cases. The IMPACT core model and the IMPACT extended model had AUCs of 0.80 (95% CI, 0.77–0.84) and 0.79 (95% CI, 0.75–0.83), respectively, for predicting mortality and 0.83 (95% CI, 0.80–0.86) and 0.84 (95% CI, 0.81–0.88), respectively, for predicting unfavorable outcomes for the 607 patients in the PLANC cohort. When applying the IMPACT lab model, the discrimination improved with an AUC of 0.87 for both mortality (95% CI, 0.84–0.91) and unfavorable outcomes (95% CI, 0.84–0.92). The Helsinki CT clinical model showed very similar discriminative ability for mortality (AUC 0.80, 95% CI 0.76–0.83) and unfavorable outcome (AUC 0.86, 95% CI 0.83–0.89). The Helsinki CT clinical model showed comparable discrimination with the IMPACT core and extended model for predicting 6-month mortality. The IMPACT lab model had a significantly higher AUC than the Helsinki CT clinical model for predicting 6-month mortality (AUC 0.87 vs. AUC 0.80, *p* < 0.001). Conversely, the Helsinki CT clinical model had significantly higher AUC values for predicting 6-month unfavorable outcomes than the IMPACT core and extended models (AUC 0.86 vs. AUC 0.83–0.84, *p* < 0.05 for both). However, the AUC values for the Helsinki CT clinical model and the IMPACT lab model were comparable for predicting unfavorable outcomes (AUC 0.86 vs. AUC 0.87, *p* > 0.05).

**Table 4:** Validation of IMPACT models and Helsinki CT clinical model for prediction of 6-month mortality and unfavorable outcome in PLANC cohort.

**Models**	**6-month mortality**				**6-month unfavorable outcome**			
	**AUC^a^**	**slope**	**calibration-i.t.l**.	**R^2b^**	**AUC^a^**	**slope**	**calibration-i.t.l**.	**R^2b^**
IMPACT core model	0.80	1.02	−0.75	0.30	0.83**	1.09	−0.25	0.41
IMPACT extended model	0.79	0.98	−0.77	0.29	0.84*	1.17	−0.51	0.45
IMPACT lab model	0.87**	1.56	−0.90	0.47	0.87	1.31	−0.47	0.50
Helsinki CT clinical model	0.80	0.73	−0.29	0.31	0.86	0.99	−0.29	0.49

a. AUC, area under the curve,

b Nagelkerke R^2^

* p-value difference in AUC IMPACT and Helsinki model < 0.05,

** p < 0.001 comparison AUC of Helsinki and IMPACT model.

Calibration-i.t.l., Calibration in the large.

#### Calibration

**Figure 2:**
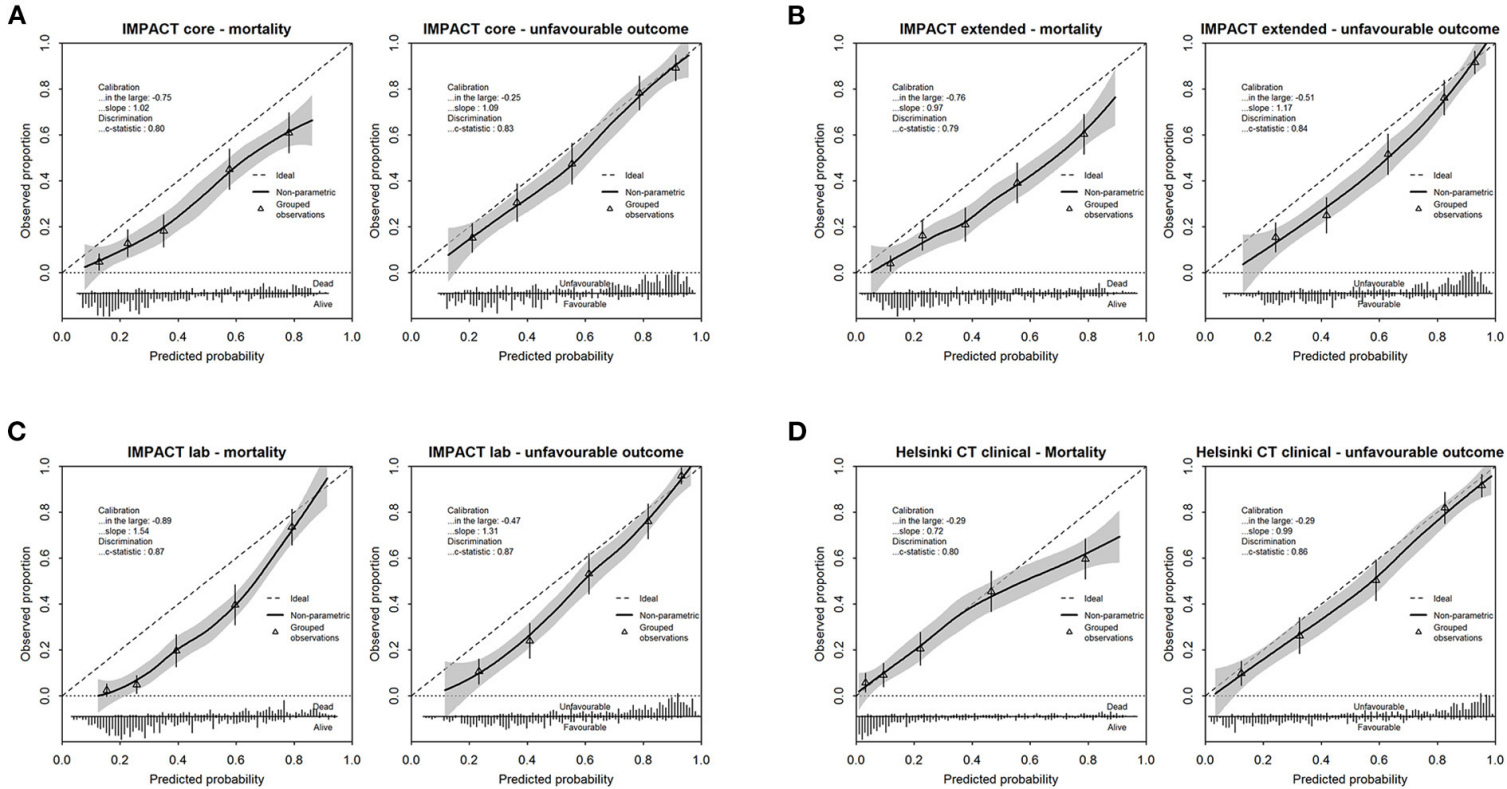
Calibration plots depicting observed vs. predicted outcome for mortality and unfavorable outcome for the IMPACT models [core model **(A)**, extended model **(B)**, lab model **(C)**] and Helsinki CT clinical model **(D)**. Performance parameters (calibration in the large, slope and c-statistic) are displayed in each calibration plot. The frequencies of predicted probabilities, differentiated for the observed outcome of interest, is presented in the lower bar graph.

The calibration plots showed a reasonable agreement between observed and predicted outcomes for all models, which was generally better for the prediction of unfavorable outcomes (Figure 2). Overestimation was, however, substantial for the IMPACT models. The observed frequencies of unfavorable outcomes and death were lower than those predicted in all cases for the IMPACT models (calibration-in-the-large < 0). The calibration slopes of the IMPACT core and extended model were close to one, while the predictor effects of the IMPACT lab model were on average too weak. The calibration slope of the Helsinki CT clinical model was well-below one, indicative of predictive risks that were too high. The calibration slope of the Helsinki CT clinical model predicting an unfavorable outcome was close to one.

When predicting 6-month mortality, the IMPACT lab model showed the highest Nagelkerke *R*^2^ (0.47). The *R*^2^ values of the other IMPACT models and the Helsinki model were comparable, with values of ~0.30. The *R*^2^ values when predicting an unfavorable outcome were highest for the IMPACT lab model and Helsinki model (0.50 and 0.49) and lower for the IMPACT core and IMPACT extended model (0.41 and 0.45, respectively).

## Discussion

Prognostication is important, especially when considering a potentially life-saving but not necessarily restorative surgical intervention (20, 21). Uncertainty, however, exists if prediction models developed in broad populations and other settings may apply to the specific population of surgical patients with TBI, of whom a large number underwent decompressive craniectomy. This external validation study demonstrated that both the IMPACT and the Helsinki CT clinical model provided adequate to good prediction of 6-month mortality and outcome in surgical patients with TBI, consistent with other validation studies (7, 11–15). Notably, significant differences did exist between model iterations in predictive performance. The more complex IMPACT models and the Helsinki CT clinical model consistently provided greater predictive power than the IMPACT core model. The use of CT predictor variables can explain the superiority. We found that all the models suffered from an overestimation of 6-month outcome risk (i.e., the models' predicted risk of poor outcome was higher than the observed frequency of poor outcome). We considered several potential causes contributing to the observed differences in model performance: case mix and treatment, predictor effects and geographical setting.

### Case-mix and therapy

The population we focused on was very different from that on which the IMPACT models were developed but more comparable to the Helsinki population. In particular, mass lesions were more frequent in PLANC than in IMPACT (70 vs. 38%). In the Helsinki cohort, a mass lesion was recorded in 60% of cases. This difference may largely explain the poor calibration intercept of the IMPACT models compared to the Helsinki CT clinical model. The second major difference relates to the rate of surgery. In the PLANC cohort, all patients underwent surgical therapy. In the IMPACT studies, the rate of surgery was substantially lower and varied, being 37 to 39% in the observational studies underpinning IMPACT and 11–38% with a median of 27% in the randomized clinical trials. In the Helsinki cohort, the rate of surgery was 35%. More than 85% of our patients underwent a decompressive craniectomy following the development of intractable intracranial hypertension or evacuation of an intracranial hematoma (Table 5). Miscalibration of the CRASH model in patients undergoing a decompressive craniectomy has previously been reported by Honeybull et al. (22). In a cohort of 270 patients undergoing decompressive craniectomy, they – like us – found excellent discriminatory ability but poor calibration. An interaction between the effectiveness of decompressive craniectomy and the severity of TBI should perhaps be considered and may explain the higher rate of unfavorable outcomes (74%) in patients who had undergone bilateral decompression (*n* = 82) compared to those with unilateral decompression (53%; *n* = 449). The poor outcome in patients with bilateral decompression (diffuse injury) is consistent with the results of the DECRA study (23).

**Table 5:** Differences in surgical types were examined as a potential explanation for outcome in PLANC cohort.

**Surgical types**		**6-month outcome**, ***n*** **(%)**	
	**Total**	**Dead** **(*n* = 170)**	**Unfavorable outcome** **(*n* = 315)**
Unilateral decompressive craniectomy	449	129 (29%)	239 (53%)
Bilateral decompressive craniectomy	82	37 (45%)	61 (74%)
Posterior fossa hemicraniectomy	8	4 (50%)	5 (63%)
Craniotomy^a^	68	0	10 (15%)

a craniotomy for evacuating mass lesion (e.g, subdural hematoma, intracerebral hemorrhage or epidural hematoma) without performing a decompressive craniectomy.

### Predictor effects

Predictor effects were different in the validation data. Compared to the IMPACT cohort, specifically the prognostic strength of a low GCS motor score (no response or extension) and non-reactive pupillary responses were much stronger, while EDH and tSAH were smaller in the PLANC cohort (Table 3). The substantially stronger effect of an absent motor score is likely explained by the fact that in our region, only a few patients are sedated and intubated at the scene of the accident. As a consequence, an absent motor score accurately reflects a poor neurological condition without being influenced by the effects of sedation. A false low GCS score has been reported in 13% of patients with severe TBI (24). Miscalibration might also occur because of differences in the scoring of GOS between settings; these changes may influence both the outcome distribution and predictor effects and lead to miscalibration (7).

### Geographic setting

The different geographic settings of PLANC recruitment with enrollment of exclusively Asian patients may also be a relevant factor. In Asian patients, poorer outcomes were found, but the numbers were too small to permit definitive conclusions. To date, it is uncertain whether these differences may be related to genetic constitution or possibly result from disparities in treatment. Differences in outcomes related to geographic location have previously been identified in the CRASH trial (4).

### Perspective

We consider that differences in case-mix and in predictor effects have mainly contributed to the poor calibration of the models. It would appear unlikely that geographic setting or race were of major influence, as we found a better than predicted outcome, which would be opposite to the effects attributed to race. Despite substantial differences in baseline characteristics between the PLANC and Helsinki cohorts, the proportion of patients with a mass lesion was much more comparable between these cohorts than between PLANC and IMPACT. This better comparability may explain the better calibration of the Helsinki CT clinical model. The high discriminative performance of both models confirms the validity of the predictors. Nevertheless, the presence of miscalibration suggests the need to recalibrate models when used in very different settings than the development population. As the standards of care advance and the treatment results improve, it is only to be expected that the calibration of existing models will change. Recalibration or updating over time is advocated.

### Strengths and limitations

Several limitations of our study should be acknowledged. First, the validation population concerns a selected population with a different case mix compared to the development populations. Second, it was performed in different settings. These limitations may, however, be considered strengths, as they address generalizability. Third, CT assessments were performed by a single expert, and intraobserver agreement was not determined. Fourth, it is a single center setting, which makes inadequate calibration impossible to distinguish from center level effects. This may have induced some bias in scorings, but if present these would be consistent.

## Conclusions

The IMPACT and Helsinki clinical models for predicting outcome in patients with moderate to severe TBI showed excellent discrimination upon external validation on a large cohort of surgically treated patients, of whom 87% underwent a decompressive craniectomy. On calibration, all models showed some overestimation with a better than predicted outcome. These findings confirm the validity of predictors but suggest a need to recalibrate models to specific settings.

## Data availability statement

The raw data supporting the conclusions of this article will be made available by the authors, without undue reservation.

## Ethics statement

The study protocol was approved by the Renmin Hospital of Wuhan University and Anhui Medical University Affiliated Wuxi Clinical College Clinical Research Ethics Committee (YXLL-2022019). Written informed consent was obtained from the individual(s) for the publication of any potentially identifiable images or data included in this article. Written informed consent for participation was not required for this study in accordance with the national legislation and the institutional requirements.

## Author contributions

LC, YW, and HX were involved in the conception and design of the study. JH and CZ were involved in the data analysis. AM, HS, and DN were involved in the acquisition of data. RR and AM contributed substantially to drafting the manuscript and figures. All authors read and approved the final manuscript.

## Conflict of interest

The authors declare that the research was conducted in the absence of any commercial or financial relationships that could be construed as a potential conflict of interest.

## Publisher's note

All claims expressed in this article are solely those of the authors and do not necessarily represent those of their affiliated organizations, or those of the publisher, the editors and the reviewers. Any product that may be evaluated in this article, or claim that may be made by its manufacturer, is not guaranteed or endorsed by the publisher.
