# Supplementary material for: Performance of the IMPACT and Helsinki models for predicting 6-month outcomes in a cohort of patients with traumatic brain injury undergoing cranial surgery
Source: Front Neurol. 2022 Oct 31;13:1031865. doi: 10.3389/fneur.2022.1031865 (PMC9659637; doi:10.3389/fneur.2022.1031865)
Supplement: Supplementary file 1 [file Data_Sheet_1.docx]

| **Appendix 1: GLASGOW outcome assessment method employed by SyNAPSe trial**  **Q1: Has the subject resumed pre-injury occupational status?**  **0=No**  **1= D Subject has resumed household responsibilities**  **2= C Subject has resumed academic pursuits**  **3= B Subject could have resumed occupational status, but currently has a reduction in performance level due to outlying circumstances that are not brain related**  **4= A Subject has resumed pre-injury employment**  **Q2: Has the subject resumed at least half as often as the pre-injury level of social activities?**  **0=No**  **1= Yes**  **Q3: Has the subject resumed pre-injury level of independence in activities of daily living?**  **0= No**  **1= Yes**  **Q4: Is the subject able to be left alone at home and is able to care for her/himself at the same level of pre-injury?**  **0= No**  **1= Yes**  **Q5: Has the subject resumed independence in transportation?**  **0= No**  **1= Yes**  **Q6: Does the subject respond to verbal communication and/or obey commands?**  **0= No**  **1= Yes**  **GOS rating**  **- Good recovery**  **- Moderate disability**  **- Severe disability**  **- Vegetative state**  **RATING THE GOS:**  **Good recovery: if you answered YES to all questions**  **Moderate disability: if you answered NO to Q1 + Q2 and YES to Q3 + Q4 + Q5 + Q6**  **Severe disability: if you answered NO to Q3, Q4 OR Q5, and YES to Q6**  **Vegetative state: if you answered NO to Q6**  **Table S1:** Summary of missing values.   \| **Variable name** \| **N of missing values** \| **% of missing values** \| \| --- \| --- \| --- \| \| Pupils \| 5 \| 0.82% \| \| Hypoxia \| 8 \| 1.32% \| \| Hypotension \| 2 \| 0.33% \| \| Glucose \| 31 \| 5.11% \| \| Hb \| 6 \| 0.98% \| \| Motor score \| 2 \| 0.33% \| |
| --- | --- | --- | --- | --- | --- | --- | --- | --- | --- | --- | --- | --- | --- | --- | --- | --- | --- | --- | --- | --- | --- |

| **Table S2.** Associations between predictors and 6-month outcome in PLANC, IMPACT and Helsinki cohorts. | | | | | | | | | | |
| --- | --- | --- | --- | --- | --- | --- | --- | --- | --- | --- |
| **Characteristics** | **Coding** | **6 Month Outcome Number(%)** | | | | | | | | |
|  |  | **PLANC（n=607）** | | | **IMPACT （n=8509）** | | | **Helsinki （n=869）** | | |
|  |  |  |  |  |  |  |  |  |  |  |
|  |  | **Total** | **Dead（n=170）** | **Unfavorable（n=315）** | **Total** | **Dead（n=2396）** | **Unfavorable（n=4082）** | **Total** | **Dead** | **Unfavorable** |
|  |  |  |  |  |  |  |  |  | **(n=219)** | **（n=414）** |
| **Motor score**^a^ | None (1) | 84 | 55 (65%) | 76 (90%) | 1395 | 625 (45%) | 894 (64%) | 141 | 76 (54%) | 104 (74%) |
|  | Extension (2) | 104 | 56 (54%) | 89 (86%) | 1042 | 496 (48%) | 807 (77%) | 42 | 21 (50%) | 34 (81%) |
|  | Abnormal flexion (3) | 83 | 21(25%) | 52 (63%) | 1085 | 326 (30%) | 619 (57%) | 36 | 16 (44%) | 25 (69%) |
|  | Normal flexion (4) | 28 | 9 (32%) | 20 (71%) | 1940 | 411 (21%) | 800 (41%) | 94 | 22 (23%) | 46 (49%) |
|  | Localizes(5) | 229 | 24 (10%) | 68 (30%) | 2591 | 383 (15%) | 699 (27%) | 214 | 39 (18%) | 91 (43%) |
|  | Obeys (6) | 77 | 4 (5%) | 8 (10%) |  |  |  | 342 | 45 (13%) | 114 (33%) |
| **Pupillary** | Both pupils reactive | 298 | 35 (12%) | 90 (30%) | 4486 | 790 (18%) | 1578 (35%) | 644 | 100 (16%) | 246 (38%) |
| **reactivity** | One pupil reactive | 156 | 50 (32%) | 97(62%) | 886 | 295 (33%) | 521 (59%) | 101 | 37 (37%) | 67 (66%) |
|  | No pupil reactive | 148 | 84 (56%) | 124(83%) | 1754 | 946 (48%) | 1351 (77%) | 124 | 82 (66%) | 101 (81%) |
| **Hypoxia** | Yes or suspected | 124 | 58(47%) | 92 (74%) | 1116 | 481 (43%) | 713 (64%) | 132 | 51 (39%) | 82 (62%) |
|  | NO | 475 | 110(23%) | 217(46%) | 4336 | 1158(27%) | 1928 (44%) | 737 | 168 (23%) | 332 (45%) |
| **Hypotension** | Yes or suspected | 57 | 41(72%) | 51 (89%) | 1171 | 578 (49%) | 794 (68%) | 66 | 22 (33%) | 34(62%) |
|  | NO | 548 | 128 (23%) | 263 (48%) | 5249 | 1315(25%) | 2263 (43%) | 803 | 197 (25%) | 380 (47%) |
| **Marshall CT** | I |  | - | - | 360 | 24(7%) | 50 (14%) |  | - | - |
| **Classification**^b^ | II |  | - | - | 1838 | 256 (14%) | 582 (32%) | 282 | 28 (10%) | 80 (28%) |
|  | III | 62 | 28 (45%) | 40 (65%) | 863 | 287(33%) | 456 (53%) | 37 | 12 (32%) | 18 (49%) |
|  | IV | 115 | 30 (26%) | 54 (47%) | 187 | 86(46%) | 107 (57%) | 30 | 8 (27%) | 16 (53%) |
|  | EML | 427 | 111 (26%) | 220 (52%) | 1435 | 422 (29%) | 709 (49%) | 520 | 171 (33%) | 300 (57%) |
|  | NEML |  | - | - | 509 | 217 (43%) | 293 (58%) |  |  |  |
| **tSAH**^C^ | Yes or suspected | 578 | 168 (29%) | 310 (54%) | 3313 | 1193(36%) | 1925 (58%) | 504 | 135 (27%) | 256(51%) |
|  | NO | 26 | 1 (4%) | 4 (15%) | 4080 | 724(18%) | 1462 (36%) | 365 | 84 (23%) | 158 (43%) |
| **EDH**^d^ | Yes or suspected | 145 | 17 (12%) | 44 (30%) | 999 | 207 (21%) | 358 (36%) | 90 | 10 (11%) | 19(21%) |
|  | NO | 459 | 152 (33%) | 270 (59%) | 6410 | 1794 (28%) | 3101(36%) | 779 | 209 (27%) | 71(9%) |
| ^a^Motor score was classified into five and three categories in IMPACT and Helsinki database respectively. | | | | | | | | | | |
| ^b^ Marshall CTClassification: I, no visible intracranial pathology on CT scans; II,midline shift 0-5mm;III,cistems compressed or absent with midline shift 0-5mm; IV,midline shift＞5mm, EML, any lesion surgically evacuated; NEML,high-or mixed-density lession＞25mm,not surgically evacuated.EML and NEML were combined in IMPACT and Helsinki database. | | | | | | | | | | |
| ^c^tSAH: traumatic subarachnoid haemorrhage  ^d^EDH: Epidural hemorrhage. | | | | | | | | | | |
